# New Recombinant Cold-Adapted and Organic Solvent Tolerant Lipase from Psychrophilic *Pseudomonas* sp. LSK25, Isolated from Signy Island Antarctica

**DOI:** 10.3390/ijms20061264

**Published:** 2019-03-13

**Authors:** Leelatulasi Salwoom, Raja Noor Zaliha Raja Abd. Rahman, Abu Bakar Salleh, Fairolniza Mohd. Shariff, Peter Convey, Mohd Shukuri Mohamad Ali

**Affiliations:** 1 Enzyme and Microbial Technology Research Center, Faculty of Biotechnology and Biomolecular Sciences, Universiti Putra Malaysia, Serdang Selangor 43400, Malaysia; lt.salwoom@gmail.com (L.S.); rnzaliha@upm.edu.my (R.N.Z.R.A.R.); abubakar@upm.edu.my (A.B.S.); fairolniza@upm.edu.my (F.M.S.); 2 National Antarctic Research Centre (NARC) B303, Block B, Level 3, IPS Building, University of Malaya, Kuala Lumpur 50603, Malaysia; 3 Department of Microbiology, Faculty of Biotechnology and Biomolecular Sciences, Universiti Putra Malaysia, Serdang Selangor 43400, Malaysia; 4 British Antarctic Survey, NERC, High Cross, Madingley Road, Cambridge CB3 OET, UK; pcon@bas.ac.uk; 5 Department of Biochemistry, Faculty of Biotechnology and Biomolecular Science, Universiti Putra Malaysia, Serdang Selangor 43400, Malaysia

**Keywords:** Antarctica, cold adapted lipase, *Pseudomonas* sp. LSK25, purification, organic solvent tolerant

## Abstract

In recent years, studies on psychrophilic lipases have become an emerging area of research in the field of enzymology. The study described here focuses on the cold-adapted organic solvent tolerant lipase strain *Pseudomonas* sp. LSK25 isolated from Signy Station, South Orkney Islands, maritime Antarctic. Strain LSK25 lipase was successfully cloned, sequenced, and over-expressed in an *Escherichia coli* system. Sequence analysis revealed that the lipase gene of *Pseudomonas* sp. LSK25 consists of 1432 bp, lacks an N-terminal signal peptide and encodes a mature protein consisting of 476 amino acids. The recombinant LSK25 lipase was purified by single-step purification using Ni-Sepharose affinity chromatography and had a molecular mass of approximately 65 kDa. The final recovery and purification fold were 44% and 1.3, respectively. The LSK25 lipase was optimally active at 30 °C and at pH 6. Stable lipolytic activity was reported between temperatures of 5–30 °C and at pH 6–8. A significant enhancement of lipolytic activity was observed in the presence of Ca^2+^ ions, the organic lipids of rice bran oil and coconut oil, a synthetic C12 ester and a wide range of water immiscible organic solvents. Overall, lipase strain LSK25 is a potentially desirable candidate for biotechnological application, due to its stability at low temperatures, across a range of pH and in organic solvents.

## 1. Introduction

Microbes which thrive at low temperatures (below 20 °C) are defined as psychrophilic or psychrotolerant. Globally, psychrophiles are reported as one of the most underutilized resources [1]. They are able to survive and function at low temperature by producing enzymes which have an evolved range of structural features that enables specific activity [2,3,4]. Collectively termed ‘cold-adapted’ enzymes, this study focuses on a recombinant cold-adapted lipase produced by a soil bacterium isolated from Signy Island, Antarctica.

Lipases (triacylglycerol acylhydrolase, E.C. 3.1.1.3) are able to catalyse the hydrolysis of triacyglycerols to glycerols and fatty acids at oil–water interfaces [5]. With rapid developments in the field of enzymology, cold-adapted lipases have proven advantageous in temperature sensitive applications, and most importantly for practical applications such as detergent additives, permitting effective washing in cooler water and thereby improving energy efficiency. These enzymes are also used as additives in the food industry, improving cold storage through reducing contamination and food spoilage, and as bioremediation agents in waste water treatment and the in situ bioremediation of fat-contaminated environments [6,7]. Organic solvent tolerant lipases have high potential value for enzyme-catalysed transesterification reactions for biodiesel production [8]. Cold regions, and in particular the Arctic and Antarctic, provide ideal potential sources for the isolation of novel cold-adapted lipases [9,10,11].

Increasing demand for cold adapted lipases has led to the development of recombinant lipases, whereby the lipase genes are integrated into heterologous expression host systems in order to increase the efficiency and scale of production of the desired enzyme [12]. With this background, the present study set out to isolate, purify and characterize the bacterial lipase *Pseudomonas* sp. LSK25, originally obtained from soil samples from Signy Island, Antarctica.

## 2. Results

### 2.1. Sequence Analysis and Expression of LSK25 Lipase

The lipase producing strain of *Pseudomonas* sp. LSK25 (denoted as LSK25 lipase) isolated from Signy Island, Antarctica was selected for detailed study of the expression and characterisation. The use of lipase-specific primers successfully identified the presence of a suitable lipolytic gene within this strain. Successful PCR amplification of the 1432 bp gene was followed by ligation into the heterologous expression vector pET32b (+). Similarly, a lipase-encoding marine Antarctic bacterium isolated from seawater around King George Island was also successfully expressed into vector pET22b with a lipase gene product of 1119 bp [13]. *Pseudomonas* sp. AMS8, a cold-adapted organic solvent tolerant lipase isolated from Casey station, Antarctic was also successfully ligated into vector pET32b (+) with an amplified lipolytic gene of 1432 bp [14]. Another Antarctic cold-adapted lipase *Pseudomonas* sp. AMS3, which has the ability to show lipolytic activity across a broad pH profile, was also expressed in the Plasmid for Expression by T7 RNA polymerase (pET) system with a ligated lipase gene product of 1353 bp [15].

**Figure 1:**
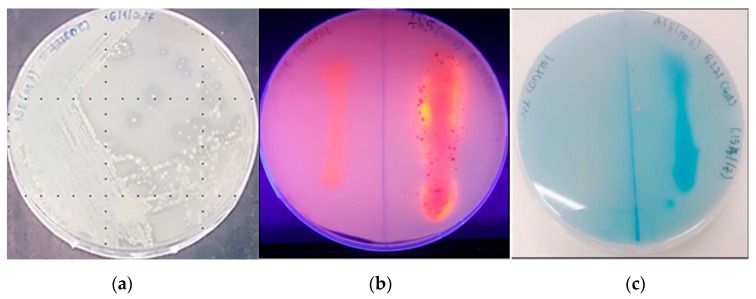
Qualitative analysis of recombinant LSK25 lipase on various agar plates. (**a**) clearing zone on tributyrin agar; (**b**) lipolytic activity on Rhodamine B, with substrate hydrolysis leading to the formation of an orange fluorescent halo upon exposure to UV radiation; and (**c**) lipolytic activity confirmation due to dye indicators on triolein agar. Negative control is indicated in the qualitative plates of **b** and **c**.

**Figure 2:**
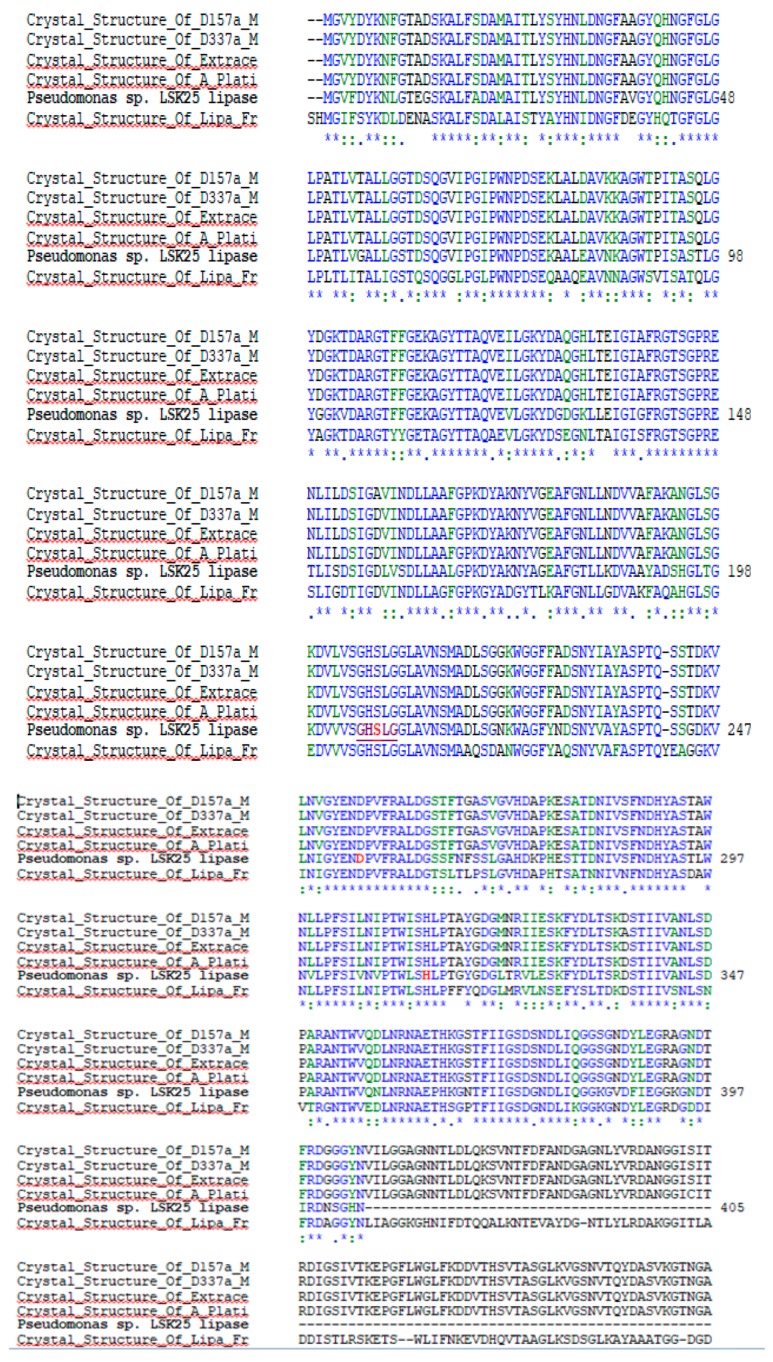

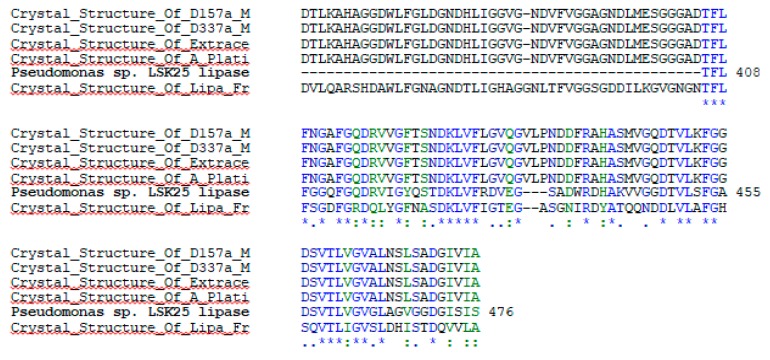
Alignment of the LSK25 lipase sequence with Pseudomonas lipases of known three-dimensional structure. Red highlight represents the catalytic triad: Ser^207^, Asp^255^, His^313^. Purple highlight underlined represents pentapeptide: GHSLG (GXSXG), (*) blue represents conserved residue; (:) green represents conservation of strong groups; (.) blue represents conservation of weak groups, absence of nucleotide for alignment (-). LSK25 lipase amino acid sequences were aligned with amino acid sequences of the crystal structures of D157A mutant of *Pseudomonas* sp. MIS38 lipase, D337A mutant of *Pseudomonas* sp. MIS38 lipase, extracellular lipase from *Pseudomonas* sp. MIS38, platinum-bound S445C mutant of *Pseudomonas* sp. MIS38 lipase [16,17] and LipA from *Serratia marcescens* [18].

Qualitative screening of LSK25 lipase showed positive evidence of lipase production. The strain was able to produce clearing zones in tributyrin agar, intense blue discolouration (triolein plates) and form orange fluorescent halos (Rhodamine B) within 48 h incubation at 4 °C (Figure 1). Sequence analysis of the LSK25 lipase revealed an open reading frame (ORF) of 1432 nucleotides encoding a 476-amino acid peptide. The nucleotide and protein sequences of LSK25 lipase have been deposited in GenBank under the accession numbers MK075242 and AZL87721 respectively. The predicted molecular mass of putative LSK25 lipase was 50 kDa. Alignment with known lipases of solved crystal structures [16,17,18] identified a conserved region typical of lipase, containing the pentapeptide (GXSXG) sequence and the enzyme catalytic triad made up of Ser, Asp and His (Figure 2). The gene had 58% G+C content, absence of cysteine residue, and the coded protein lacked a signal peptide. Query on Basic Local Alignment Search Tool (BLAST) search for protein sequence similarity showed that LSK25 lipase was closely related (98% similarity) to AMS8 lipase, a well-documented cold-adapted lipase obtained near Casey station, Antarctica [19], and had 88% similarity to *Pseudomonas* sp. KB700A, a low temperature lipase of the subterranean environment [20]. Although the total numbers of negatively charged (Asp+Glu) and hydrophobic residues were similar to AMS8 lipase [19], the number of positively charged residues (Arg+Lys) and polar residues were higher.

### 2.2. Expression and Purification of Recombinant LSK25 Lipase

LSK25 lipase was cloned and expressed using the *E. coli* BL21(De3)/pET32b(+) expression system with a histidine tag. The recombinant protein showed highest lipase activity as a refolded protein from inclusion bodies indicating successful refolding of solubilized LSK25 lipase. The recombinant LSK25 protein was overexpressed at the following optimised parameters: incubation temperature 25 °C, induction time 16 h, and Isopropyl β-D-1-thiogalactopyranoside (IPTG) concentration 0.1 mM at 150 rpm.

**Figure 3:**
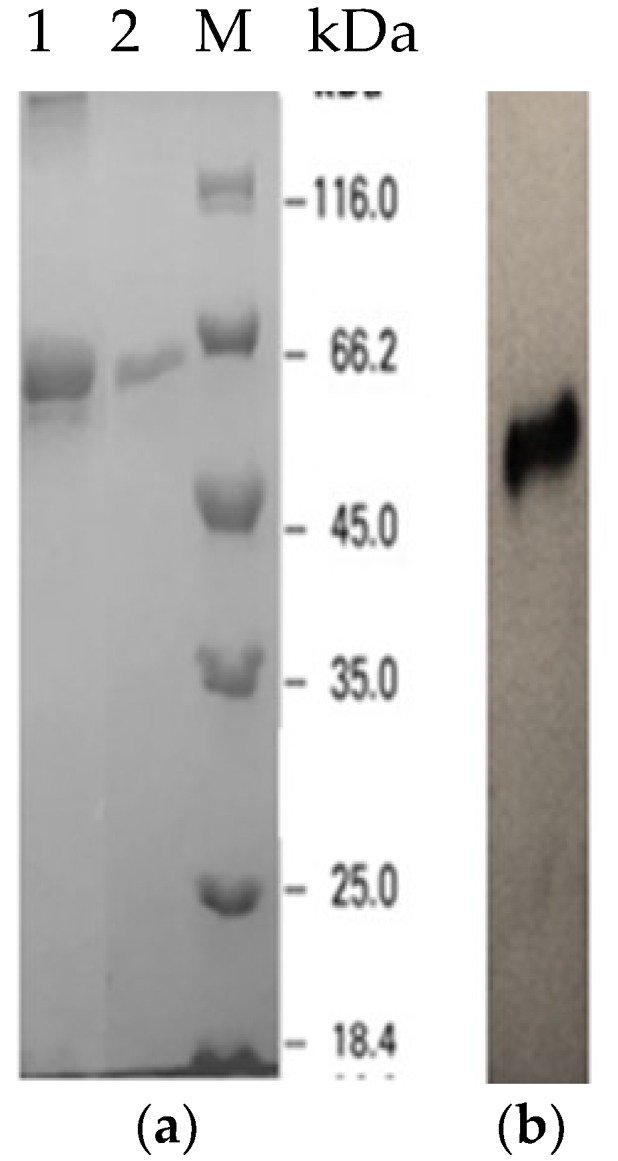
(**a**) Analysis of purified product on SDS-PAGE of LSK25 lipase. Lane 1: Refolded crude enzyme, Lane 2: Purified lipase LSK25 lipase via Ni-Sepharose affinity chromatography (65 kDa), Lane M: Standard protein marker. (**b**) native-PAGE analysis of the purified lipase LSK25.

**Figure 4:**
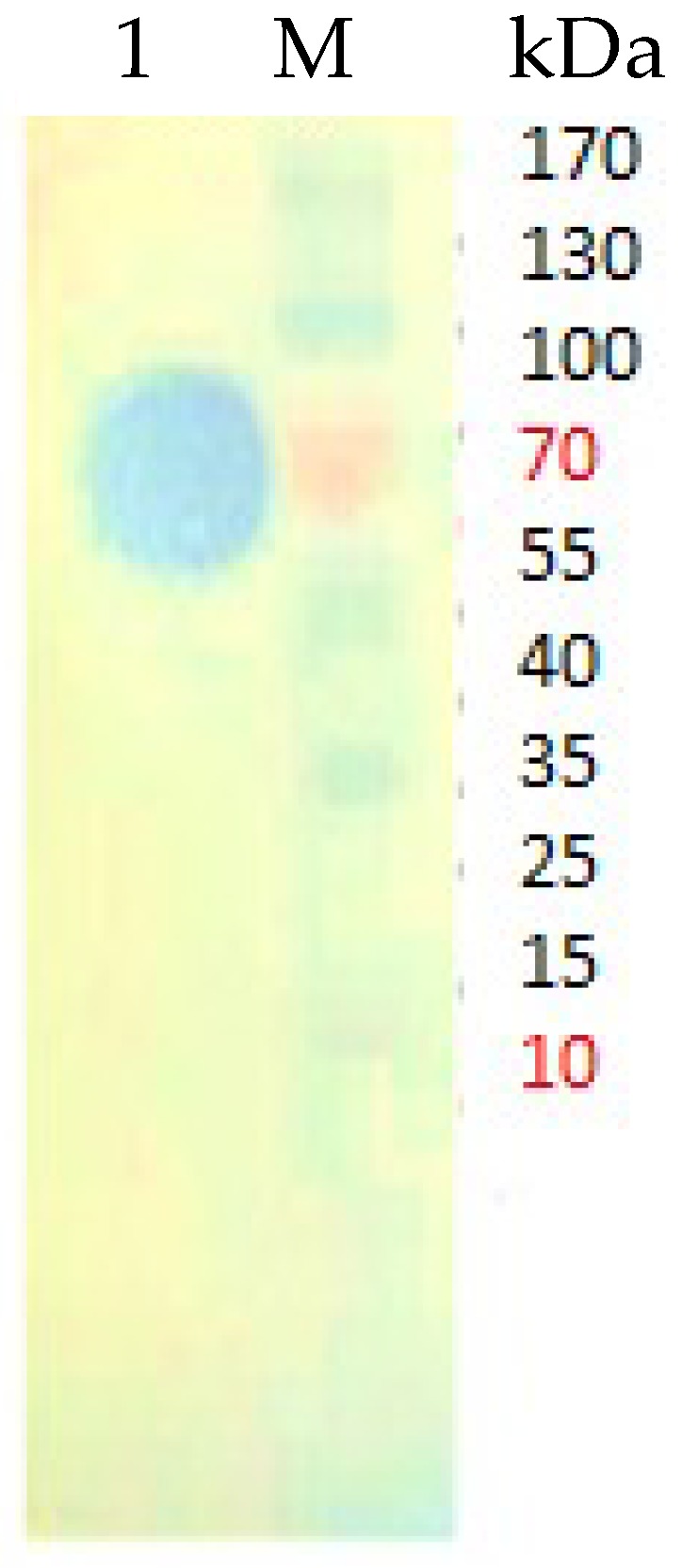
Lane 1: Activity staining with halo zone on purified LSK25 lipase. Lane M: Prestained protein ladder (Thermo Scientific, Waltham, MA, USA); red band ~10, 70 kDa; blue band ~15–55, 100–170 kDa.

The expressed refolded protein was purified using single-step Ni-Sepharose affinity chromatography. Bound protein was detected at an imidazole concentration of 300 mM, and fractions from the elution were subjected to colorimetric lipase assay to determine the presence of the target protein. Protein yield was around 44% with an overall purification factor of approximately 1.30-fold. Sodium dodecyl sulfate polyacrylamide gel electrophoresis (SDS-PAGE) indicated a single homogenous protein band at approximately 65 kDa (Figure 3a), consistent with the size of LSK25 lipase (50 kDa) merged with the fusion protein (15 kDa) from the pET-32(b)+ vector. Besides the quantitative lipase activity measurement, activity staining was carried out to ensure the target protein was biologically active. A clearing zone (halo) surrounding the target protein band on tributyrin plates was observed prior to incubation (Figure 4). The purity level of LSK25 lipase was also determined via native PAGE analysis, generating a distinctive single band (Figure 3b).

#### Dynamic Light Scattering Profile of LSK25 Lipase

**Figure 5:**
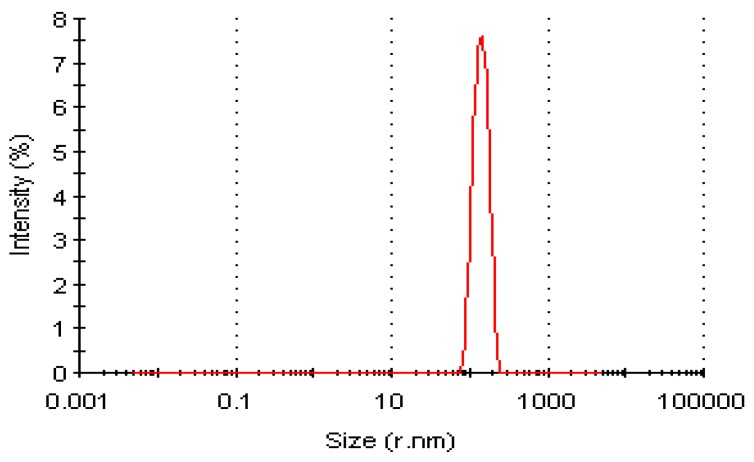
DLS profile of purified lip LSK25 at 25 °C, a single sharp peak indicating the monodispersity of the protein molecule.

Purified protein prior to native-PAGE analysis was also examined using Dynamic Light Scattering (DLS) at the optimum stability temperature of 25 °C. DLS was used to study protein aggregation in solution and in this study was used to determine the monodispersity and the homogeneity of the protein molecules [21]. A single peak was generated from purified LSK25 lipase with 22% polydispersity index (Figure 5). A polydispersity index of <0.7 further suggested the protein to be homogeneous. This result indicates that single-step purification is appropriate for LSK25 lipase.

### 2.3. Characterization of Purified LSK25 Lipase

#### 2.3.1. Effect of Temperature on LSK25 Lipase Activity and Stability

**Figure 6:**
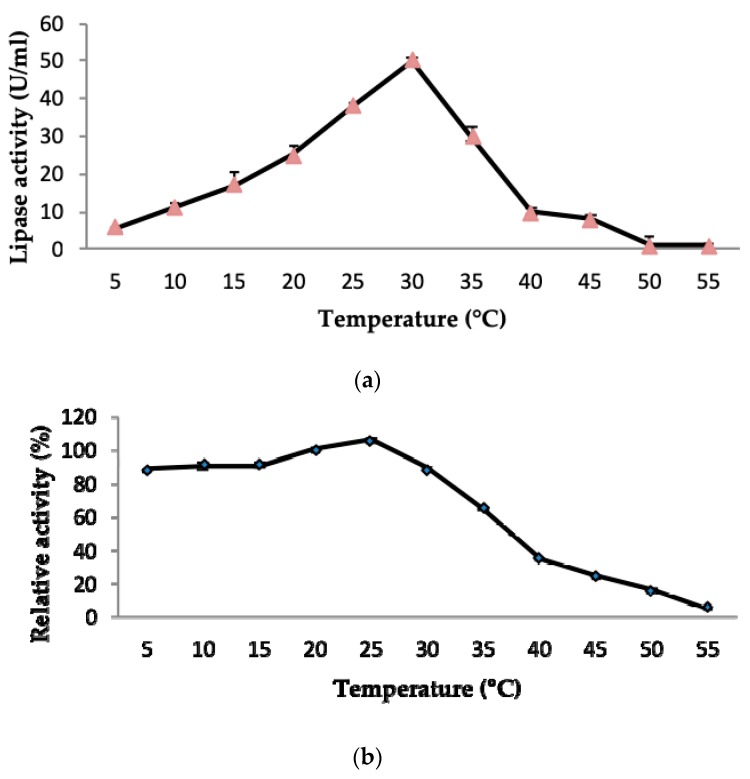
(**a**) Effect of temperature on purified LSK25 lipase activity. The lipase assay was carried out using olive oil as a substrate. Maximum lipase activity was observed at 30 °C. (**b**) The effect of temperature on purified LSK25 lipase stability. The enzyme was incubated at various temperatures for 30 min and the assay was performed at the optimum temperature. Activity was calculated relative to 25 °C as 100%. Error bars represent standard deviation (*n* = 3).

Cold-adapted enzymes can be extremely sensitive to any significant alteration in temperature. In this study the effect of temperature on the lipolytic activity and thermal stability of LSK25 lipase was measured over a temperature range between 5 to 55 °C. Maximum lipolytic activity was recorded at 30 °C (Figure 6a) at about 50.5 U/mL and a sharp decline was observed from 40 °C onwards with lipase activity dropping below 10 U/mL. Almost no activity was recorded at temperatures of 50–55 °C. The protein stability was 100% of relative activity at 25 °C, with at least 50% relative activity achieved from 15 °C onwards (Figure 6b). Temperatures higher than 30 °C led to a rapid reduction in LSK25 lipase activity.

#### 2.3.2. Effect of pH on LSK25 Lipase Activity and Stability 

**Figure 7:**
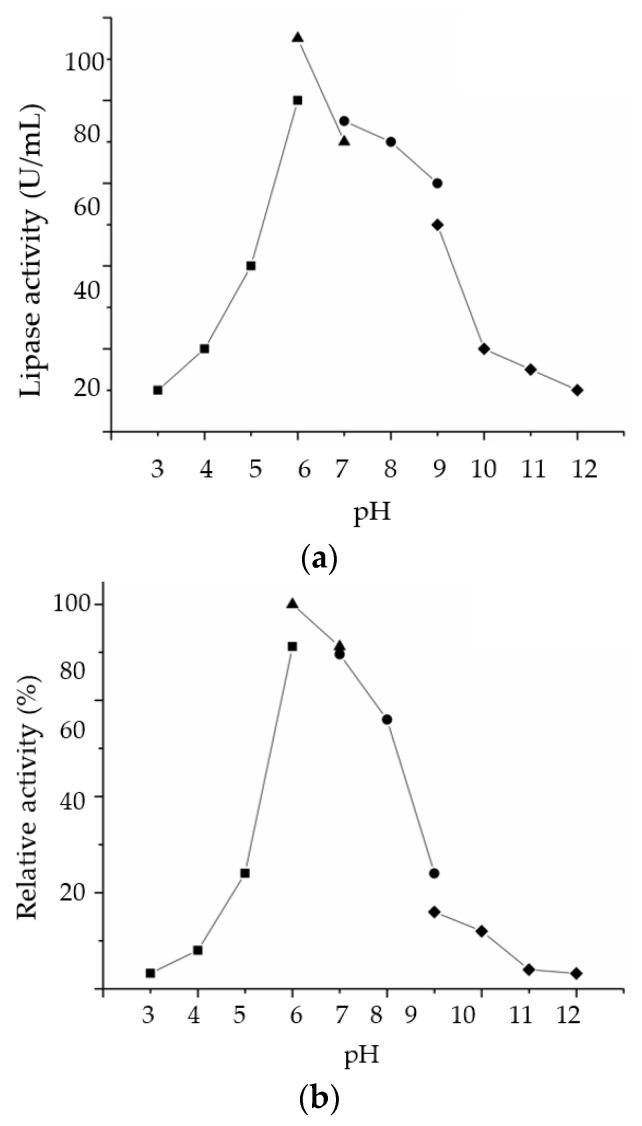
(**a**) Effect of pH on purified LSK25 lipase activity. Effect of pH on purified LSK25 lipase stability is shown in (**b**). The enzyme was pre-incubated in the different buffers for 30 min. The lipase assay used olive oil emulsion as substrate and took place at 30 °C for 30 min. Activity was calculated relative to pH 6 as 100%. The buffer systems used were: sodium acetate (pH 3–6) (filled square); potassium phosphate (pH 6–7) (filled triangle); Tris-HCl (pH 7–9) (filled circle); glycine-OH (pH 9–12) (filled diamond). Error bar represents standard deviation (*n* = 3). Absence of bar indicates the error smaller than symbols.

pH plays an important role in enzyme stability through its effects on protein tertiary structure [22]. Optimum pH and stability of LSK25 were tested across a pH range of 3–12. Gradual increases in the stability and activity were recorded from pH 3–6, with reductions at pH > 7. Highest lipolytic activity was recorded in potassium phosphate buffer followed by sodium acetate, recording lipase activity of 88.2 and 81.3 U/mL, respectively, at pH 6. Extreme acidic (pH 3) and alkaline (pH 12) conditions were not favorable for lipolytic activity of LSK25 (Figure 7a). The enzyme was active across a broad range of pH (pH 6–8), retaining more than 60% of activity relative to that at pH 6 (potassium phosphate), when the highest activity was recorded. The enzyme was most active at a slightly acidic to neutral pH 6–7, and was most inhibited in acidic conditions of pH 3–5 and alkaline conditions of pH 9–12 (Figure 7b).

#### 2.3.3. Effect of Metal Ions on LSK25 Lipase Activity

**Figure 8:**
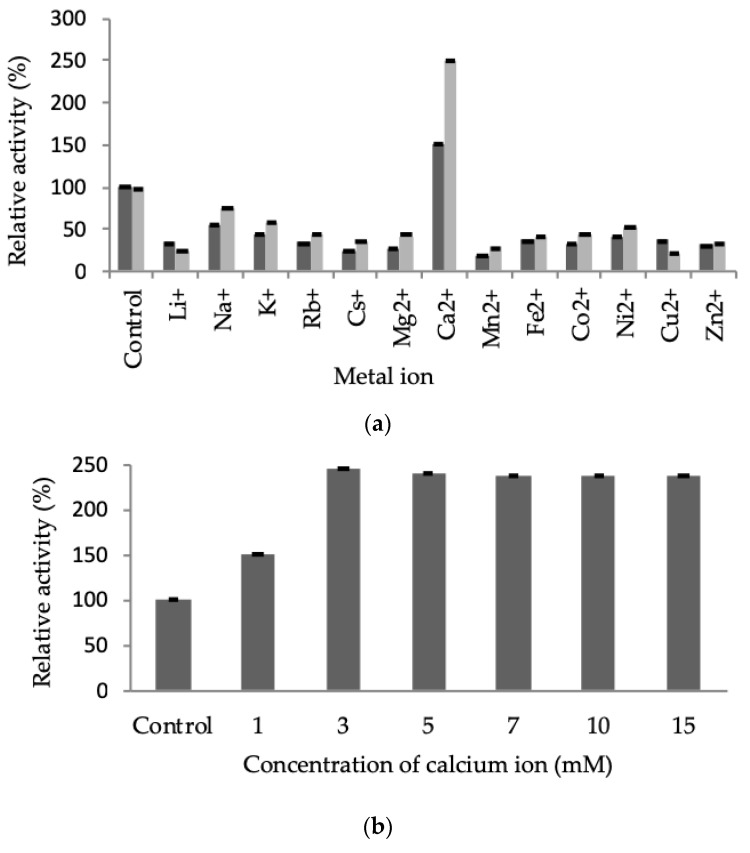
(**a**) Effect of metal ions on LSK25 lipase activity. All assays were carried out using olive oil as substrate. The enzyme was pre-incubated at 25 °C with various metal ions at concentrations of 1 mM (black) and 5 mM (grey). (**b**) Effect of concentration of calcium ions (1–15 mM). The enzyme was pre-incubated at 25 °C for 30 min prior to lipase assay. The assays took place at 30 °C for 30 min. The control consisted of the absence of metal ions), with activity expressed relative to the control value. Error bars represent standard deviation (*n* = 3).

Specific metal ions are often involved in maintaining active site structure and enhancing catalytic activity in enzymes [23]. In this study the influence of 13 metal ions on lipolytic activity was tested at concentrations of 1 and 5 mM. Activity was determined relative to control conditions in the absence of any metal ions (Figure 8a). Lipase LSK25 activity was strongly suppressed in the presence of all metal ions other than Ca^2+^. Maximum activity of 200–250% relative to the control was recorded in the presence of the Ca^2+^ ion. LSK25 was also tested at Ca^2+^ ion concentrations from 1–15 mM, demonstrating that a minimum concentration of 3 mM was sufficient to obtain the maximum enzyme activity (Figure 8b).

#### 2.3.4. Effect of Natural Oil and Ester Substrates

**Figure 9:**
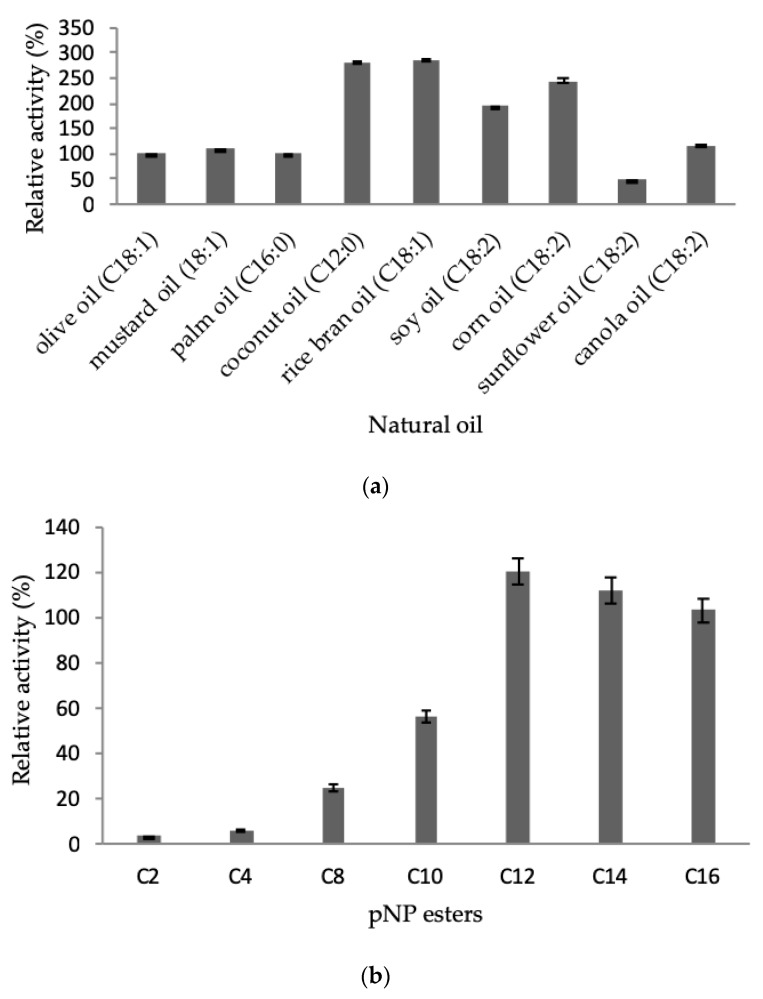
(**a**) Substrate specificity of purified LSK25 lipase toward various natural oils. (**b**) Substrate emulsion prepared with various synthetic esters. Incubation took place over 30 min at 25 °C and the assay was performed at 30 °C. Data shown are the means from triplicate measurements expressing activity relative to that with (**a**) olive oil and (**b**) *p*-nitrophenyl. Error bars represent standard deviations of means (*n* = 3).

Each enzyme has unique substrate specificity. LSK25 lipase demonstrated excellent affinity towards long-chain triglycerides (C12–18) for natural oil and ester substances. LSK25 recorded the highest activity in both coconut oil (C12:0) (291%) and rice bran oil (C18:1) (297%), relative that of the control (olive oil). A doubling of activity was also observed for soy oil (C18:2) (200%) and corn oil (C18:2) (240%) (Figure 9a). Mustard oil (C18:1), palm oil (C16:2) and canola oil (C18:2) gave similar activity to that of olive oil (C18:1) as the control substance. However, a 50% decrease in lipolytic activity was observed using sunflower oil (C18:2). When provided with ester substrates, LSK25 showed highest activity on *p*-nitrophenyl laurate (C_12_) followed by myristate (C_14_) and palmitate (C_16_), again supporting the preferential activity of this cold-adapted lipase on long chain fatty acids (Figure 9b).

#### 2.3.5. Effect of Organic Solvents on Lipase Activity

**Figure 10:**
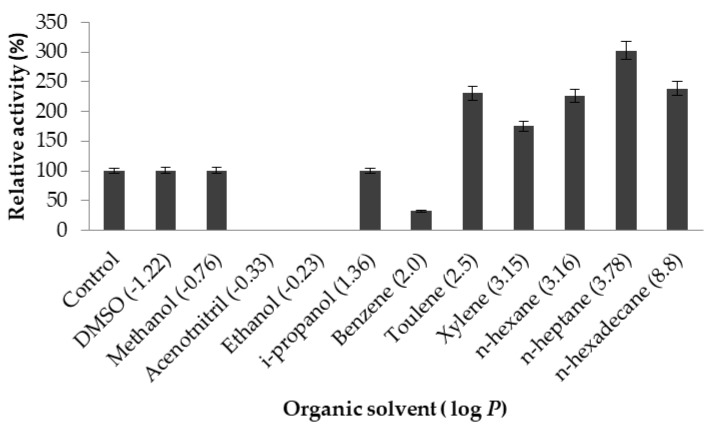
Effect of solvents on purified LSK25 lipase. The assay was carried out using olive oil as a substrate. Incubation time for the treated enzyme with organic solvent was 30 min, at 25 °C and assay at 30 °C. Remaining lipase activity was expressed relative to that of the control. Log P (water/octanol coefficient) value of each solvent are indicated in parentheses. Error bars represent standard deviations of means (*n* = 3).

Solvent polarity is an important factor in enhancing or decreasing biocatalytic stability [24]. LSK25 lipase showed elevated activity in water immiscible organic solvents, recording an almost 350% increase in activity relative to control conditions in n-heptane (log *P*_o/w_ 3.78), n-hexane (log *P*_o/w_ 3.16), n-hexadecane (log *P*_o/w_ 8.8), xylene (log *P*_o/w_ 3.15) and toluene (log *P*_o/w_ 2.5) (Figure 10). Lipolytic activity showed no significant differences between control and two other hydrophilic solvents, dimethyl sulfoxide (DMSO) (log *P*_o/w_ −1.22) and methanol (log *P*_o/w_ −0.76). Inhibition of enzymatic activity was detected using benzene (log *P*_o/w_ −2.0), which reduced activity to 75%, and complete inactivation was detected in acetonitrile (log *P*_o/w_ −0.33) and ethanol (log *P*_o/w_ −0.24).

### 2.4. Biophysical Characterisation of Purified LSK25 Lipase

#### Secondary Structure and Thermal Denaturation

**Figure 11:**
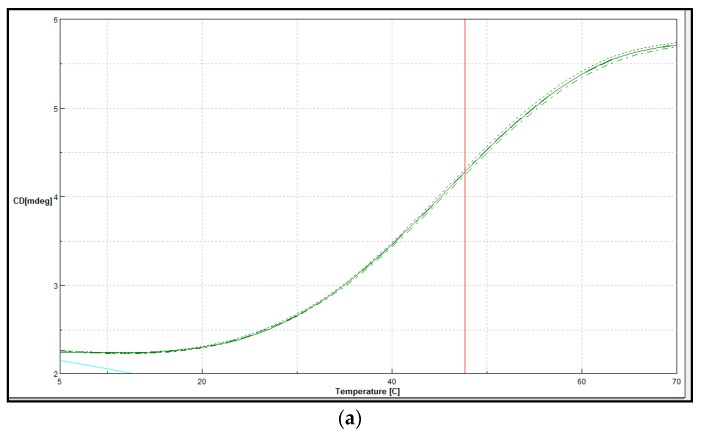

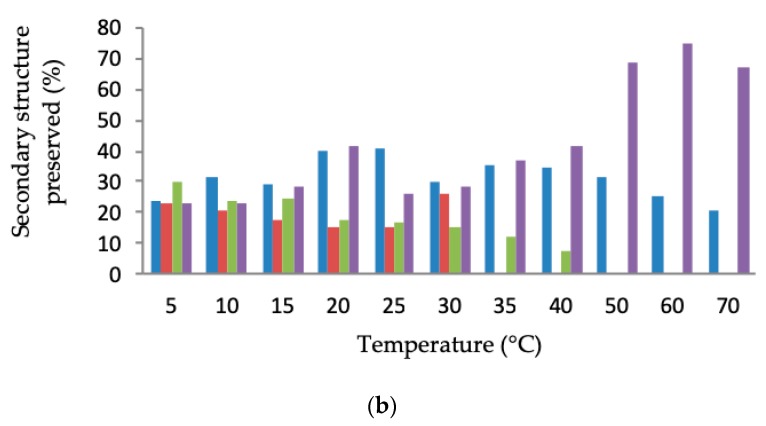
(**a**) LSK25 lipase melting point and secondary structure determination. Red vertical line indicates the melting point of LSK25 lipase when tested across a temperature ranging from 5–70 °C. Green line indicates the melting curve; blue line indicates the data background; circular dichroism milidegree (CD[mdeg]) vs. temperature. (**b**) Changes in the secondary structure of LSK25 lipase at different temperatures. Indicator: helix (blue), β-sheet (red), turn (green), random coil (purple).

A sigmoidal curve was obtained as lipase LSK25 was tested over a temperature range of 5–70 °C (Figure 11a), indicating the point of denaturation (Tm) at 47 °C, consistent with the deactivation profile as of LSK25 as shown in Figure 6b. Circular dichroism measurements have been widely used to estimate protein secondary structure [25]. Secondary structure was also tested across a similar temperature range as used in Figure 11a, with data being obtained in the 190–260 nm spectral range. Based on Figure 11b, the turn conformation increased with gradual increase in temperature from 30–35 °C. The analysis also indicated that at 35 °C lipase LSK25 lost its β-sheet. The spectrum of the α-helix then gradually decreased as temperature increased above 40 °C. Above 40 °C there was a further increase in random coil. These data are consistent with the point of denaturation as identified in Figure 11a.

## 3. Discussion

The lipase gene *Pseudomonas* sp. LSK25 obtained from Signy station, Antarctica, was expressed, purified and characterised. Cold-adapted lipases have higher flexibility and enhanced structural stability or retention of function in comparison with heat stabile lipases present in mesophiles and thermophiles [6]. These modifications aid in binding with substrates at low temperatures such as the extreme conditions of the polar regions. [1,26]. LSK25 lipase displayed several key features of the *Pseudomonas* I.3 family of enzymes as confirmed by amino acid sequence analysis. The enzyme lacks a signal peptide and cysteine residue, and has a high content of glycine residues [18,27]. These features are also prominent in other cold-adapted lipases isolated from *Pseudomonas* sp. AMS8 [19], *Pseudomonas* sp. MIS38 [13] and *Pseudomonas* sp. TK-3 [28]. The absence of the cysteine residue contributes to the flexibility of the protein structure. This feature is pivotal as, in the presence the thiolate group present in the cysteine residue, disulphide bridges could be formed which will increase the rigidity of the protein structure and hence hinder protein function at low temperatures [8,29]. The high proportion of glycine residues also contributes to the flexibility of the protein, as this amino acid does not contain any side chains that can form bonds within the protein structure [30,31]. A higher content of polar residues is also suggested as a typical feature of cold-adapted lipases, differentiating them from mesophilic counterparts [3]. Similar results were also reported by Song et al. (2008) [32] and Cieslinki et al. (2005) [33].

LSK25 lipase was cloned and expressed using the *E. coli* BL21(De3)/pET32b(+) expression system with a histidine tag, where it was abundantly expressed in the form of inclusion bodies. Inclusion bodies are often regarded as undesirable protein expression; however they can also be advantageous, through their resistance to proteolytic degradation. Inclusion bodies harbour less contaminants, which aids in reducing the number of purification steps required to recover the pure protein of interest [34,35]. Such advantages mean that recombinant proteins expressed as inclusion bodies are widely used in the commercial production of proteins [6,36]. Notably, significant numbers of cold-adapted lipases are also expressed via inclusion bodies [17,19,37,38]. Successful purification of LSK25 lipase via refolded inclusion bodies has also been reported for other cold-adapted lipases including *Acinetobacter baumannii* BD5 [37], *Psychrobacter* sp. MBP [13] and *Psychrobacter cryohalolentis* K5 [38]. As in the current study, single step Ni-sepharose chromatography purification was sufficient to obtain the pure desired protein in these studies, as also supported by the DLS results.

Temperature is a crucial factor in enzyme-catalysed reactions, therefore it is of paramount importance to determine the temperature for an enzyme to function optimally [8,39]. Higher temperatures will cause distortion of the enzyme’s active site, which can drastically reduce the activity [40,41,42]. High catalytic activities among cold-adapted enzymes have been reported at temperatures ranging from 0 to 30 °C [3,43,44,45]. LSK25 lipase recorded an optimum lipolytic activity at 30 °C, consistent with the studies of Alquanti et al. (2002) [46], Zhang et al. (2007) [47], Jiewei et al. (2014) [48], Ganasen et al. (2016) [17]. The subsequent drastic decrease in lipolytic activity at temperatures >30 °C may be due to the disruption of hydrogen bonds, ionic bonds and weak interactions within the protein molecules. Hydrogen bonds play an important role in the directional interactions that underpin protein folding, protein structure and molecular recognition [39]. LSK25 lipase can clearly be defined as a heat sensitive lipase with notable features of a cold-adapted enzyme. It has retains higher thermal stability at 25 °C in comparison to a number of well-documented cold-adapted lipases, including *Pseudomonas* AMS8 lipase [17] and the cold-adapted lipases r-LipA from *Sorangium cellulosum* [49], lipA1 from *Psychrobacter* sp. 7195 [47], r-LipA from *Pseudomonas* sp. 7323 [50] and lipX from *Psychrobacter* sp. C18 [51]. These studies also reported lower activity at temperatures of 15 °C and below. These features make LSK25 lipase a candidate for consideration in the detergent industry, as a lower temperature of 20–25 °C for washing not only increases durability of fabrics but also reduces energy consumption and is a more eco-friendly approach [4,6].

pH is crucial in maintaining the proper form of the active site in enzymes, which is composed of charged residues crucial for substrate binding. Hence any drastic changes in pH will disrupt the ionic bonds that form the enzyme structure [52]. Unlike most previously reported cold-adapted lipases, LSK25 exhibited highest stability and optimum activity at pH 6. The closest match in amino acid BLAST homology, *Pseudomonas* AMS8 lipase [17] was reported to be highly alkalophilic, while *Psychrobacter* sp. 7195 lipase [47], lipases from *Acinetobacter* sp. XMZ-26 [53] and *Yarrowia lipolytica* NCIM 3639 lipase [54] also functioned better in a more alkaline environment. *Pseudomonas gessardii* lipase [55] and *Pseudomonas reinekei* lipase [52] both showed affinity towards slightly acidic environments similar to LSK25 lipase. Optimal lipase activity and stability at slightly acidic pH can be advantageous in the oleochemical sector for the modification of triacylglycerols to improve nutritional properties in the food industry [56].

The characteristic of enhancement of lipolytic activity of LSK25 lipase in the presence of only one metal ion, Ca^2+^, has been reported in studies of other cold-adapted lipases, including *Pseudomonas stutzeri* PS59 [57], *Pseudomonas* sp. TK-3 [28], *Pseudomonas* sp. 7323 [50] and *Yarrowia lipolytica* NCIM 3639 [54]. The suppression caused by all other test metal ions may be due to alterations in the conformation of the protein to a less stable form [16]. In the presence of 5 mM Ca^2+^ activity nearly tripled in comparison to the control, consistent with the studies of Rashid et al. (2001) [20] and Tanaka et al. (2012) [28]. These authors stated that *Pseudomonas* lipases are well known to be calcium-stimulated and that some are only catalytically active in the presence of calcium ions. Testing across a range of Ca^2+^ concentrations confirmed that a minimum concentration of 3 mM was required to stimulate enhanced activity. These data confirm that LSK25 is appropriate to be incorporate into detergents, as calcium ions form complexes with ionized fatty acids which encourages the removal of free fatty acids formed in reactions at the water-oil interface. This is also economically advantageous as only a low concentration of Ca^2+^ is required for efficient activity [6,48,57].

LSK25 lipase recorded the highest lipolytic activity with coconut and rice bran oil. The use of such convenient, commercially available, and cheap substrates helps in developing cost-effective approaches towards large scale production [4]. Cold-adapted lipases from an Antarctic strain of *Bacillus pumilus* also showed similar activity with coconut oil [58]. Lauric acid, a product of coconut oil hydrolysis, is easily digested and converted into energy without any insoluble fat residue [59]. These features are also extremely useful in controlling obesity and cardiovascular diseases [60]. LSK25 was able to hydrolyse a broad range of natural oils, with an affinity towards long-chain fatty acids (C12-18), defining criteria that confirm the enzyme is a true lipase similar to other reported *Pseudomonas* lipases [18,61].

Purified LSK25 lipase displayed desirable results when treated with water-immiscible organic solvents. The activation of lipases in the presence of certain hydrophobic organic solvents can be achieved through the interactions of specific amino acid residues with the organic solvent, which enhance activity by changing the enzyme conformation from a closed to an open form [62]. Hydrophobic organic solvents possess a reduced ability to remove essential water molecules from the enzyme surface, which might underlie the increase in lipolytic activity observed in lipase LSK25 in water immiscible organic solvents. The interaction of the solvent with hydrophobic amino acid residues present in the lid/flap covering the catalytic site of the enzyme might encourage it to maintain a flexible open conformation, directly facilitating the increase in lipolytic activity [52,63]. Similar observations have been reported for lipases from *Pseudomonas stutzeri* [64] and *Pseudomonas reinekei* [52], where activity increased by between 111% and 300% when incubated in n-hexane and n-heptane. LSK25 lipase has the ability to carry out transesterification and synthesis of chiral compounds in an organic solvent environment based on the reported high activities achieved in hydrophobic solvents. LSK25 lipase can be categorised as a good candidate in the biocatalysis of esters, which may have particular application in the food flavouring industry, cosmetics and pharmaceutical sectors [8]. Thus, commercial synthesis of valuable fatty acid esters, peptide derivatives and other compounds obtained from substrates showing poor solubility in aqueous media can be achieved [4,65]. LSK25 lipolytic activity was lost in the presence of acetonitrile, ethanol and benzene. These hydrophilic solvents may distort the active site of the enzyme, thus preventing the substrate from accessing it efficiently [17]. Such inhibition has been reported in other cold-adapted lipases, including *Oceanobacillus* Strain PT-11 [48], *Streptomyces* sp. CS268 [66] and *Pseudomonas aeruginosa* LX1 [67].

Circular dichroism (CD) is widely used in identifying secondary structure and folding properties of proteins [17]. CD analysis indicated an increase in turn formation with decreasing temperature, which will help maintain the flexibility of the enzyme at lower temperature [68]. Conversely, as temperature increases, the substantial decrease in turn formation indicates that the enzyme will become more rigid, and the complete loss of structure above 40 °C correlates well with the denaturation point as indicated by CD analysis. Wang et al. (2017) [69] indicated that, as cold-adapted enzyme structure becomes more rigid, the enzyme lid will lose its function. β-sheet structure provides rigidity and acts as a backbone for the protein structure, which suggests that, at temperatures above 35 °C, the 3D structure of the protein starts to become distorted [70]. The spectrum of the α-helix gradually decreased as temperature increased above 40 °C. The α-helix is an essential element supporting the catalytic domain, and reduction in this structure above 40 °C again indicates the progressive deactivation of the enzyme [18], consistent with the measured Tm. The increase in random coil as temperature increased also can lead to destabilisation of the enzyme and changes in folding in the protein structure [17,70].

## 4. Materials and Methods

### 4.1. Bacterial Strains and Plasmids

The bacterial strain *Pseudomonas* sp. was initially isolated from soil samples collected during the austral summer of 2006/2007 around the living quarters of Signy station, Antarctica, within the South Orkney Island archipelago. The isolate was denoted as *Pseudomonas* sp. LSK25 and identified using 16S rDNA analysis to have a closest homology (98%) with *Pseudomonas* sp. An22 and *Pseudomonas* sp. JPK1, isolated from different parts of the Antarctic continent [71]. pGEMT Easy vector (Promega, Madison, WI, USA) and pET32b (Merck, Kenilworth, NJ, USA) plasmids were used for general cloning and protein expression, respectively. *E. coli* BL21(De3) was used for the expression of the recombinant LSK25 lipase.

#### 4.1.1. Qualitative Analyses

Prior to lipolytic screening the strain was grown in normal strength Luria Broth (LB) at 4 °C for 72 h under constant shaking (150 rpm). All the cultures in this study were incubated in the orbital shaker (LM-510RD, YihDer Tech. Co., Xinbei, Taiwan, China), cultured in 1 L Duran Scott bottles (Sigma Aldrich, St. Louis, MO, USA). The cultures were screened qualitatively on tributyrin agar plates’, consisting of tributyrin at 1.0% v/v. Lipolytic activity was confirmed through the formation of clear zones around the colonies. Positive isolates were then further tested for lipase production on rhodamine B (0.001% *w*/*v*), and Victoria Blue (0.01% *w*/*v*) agar plates containing tributyrin, triolein or olive oil as substrate [53]. Lipolysis was confirmed through changes in the appearance of the substrate, such as formation of a clear zone and change in color of the indicator dye used [54].

#### 4.1.2. Quantitative Analyses

Quantification of lipase activity was conducted by extracting the free fatty acid (FFA) released with isooctane and coloring with copper reagent (Kwon & Rhee, 1986) [72]. A standard curve of oleic acid was generated to permit quantification of the lipase activity. Lipase activity was assessed by a simple and rapid colorimetric method modified from Kwon & Rhee (1986), using olive oil as substrate [73]. The reaction mixture, consisting of 1.0 mL of crude enzyme, 2.5 mL olive oil emulsion (1:1 ratio of olive oil and phosphate buffer [K_2_HPO_4_ (50 mM), KH_2_PO_4_ (50 mM)] at pH 7.0) and 0.02 mL of 20 mM CaCl_2_·2H_2_O, was incubated in a water bath shaker at 4 °C for 30 min and an agitation rate of 200 rpm. The reaction was then terminated by the addition of 1.0 mL of 6 N HCl. The FFAs were subsequently extracted by the addition of 5.0 mL isooctane and vigorous mixing using a vortex mixer for 30 s. The upper isooctane layer (4 mL) containing the FFAs were transferred to a test tube containing 1 mL of copper reagent and vortexed again. The reagent was prepared by adjusting the solution of 5% (*w*/*v*) copper (II) acetate-1-hydrate to pH 6.1 with pyridine. The amount of FFAs dissolved in the isooctane layer was measured at 715 nm using a spectrophotometer (SmartSpec^TM^ PLUS BioRad, Hercules, CA, USA). One unit of lipase activity was defined as the release one micromole of free fatty acid in one min.

### 4.2. Recombinant Cold Adapted Lipase Gene

DNA manipulation was performed based on a standard method [73]. Strain LSK25 was grown in normal strength LB broth as described in Section 4.1 and genomic DNA was extracted using Qiagen^TM^ DNeasy Blood & Tissue Kits based on the manufacturer’s protocol (Qiagen, Germantown, MD, USA). The amplification of the lipase encoding gene was carried out using PCR primers from a reported cold-adapted lipase [19]. This primer set (forward 5’-ATG GGT TTT GAC TAT AAA-3’, reverse 5’-TTA GCT GAT GGA AAT TCC ATC-3’) was used to amplify a 1432 bp product. The primers were integrated with the restriction enzymes *Bam*H1 and *Xho*1 (Fermentas, Waltham, MA, USA). The purified PCR product was cloned into the pGEM^®^-T Easy (Promega, Madison, WI, USA) vector following the manufacturer’s instructions. The recombinant plasmid was extracted following the protocol provided by the GeneAll^®^ Exprep^TM^ Plasmid Quick kit (GeneAll, Seoul, Korea) and was subsequently sequenced.

### 4.3. LSK25 Lipase Sequence Analysis

Sequence data obtained were analyzed using the GenBank database BLASTp (http:// www.ncbi.nih.gov) from the National Centre of Biotechnology (NCBI) to perform an apparent homology search for the amplified lipase. Amino acid composition and molecular mass of the lipase were predicted using Expasy Tools (https://web.expasy.org/translate/). The conserved regions were analyzed via multiple sequence alignment using CLUSTALW in Biology Workbench (http://workbench.sdsc.edu). The lipase sequence was also analyzed for the presence of a signal peptide by the online Signal P 4.1 Server program (http://www.cbs.dtu.dk/services/SignalP).

### 4.4. Expression of the Lipase Encoding Gene in *E. coli* Cells

The PCR product encoding the mature lipase gene was digested with *Bam*H1 and *Xho*1 restriction enzymes and then ligated into the expression vector *E. coli* BL21(De3)/pET32b. Positive transformants were indicated by the formation of halo zones on tributyrin-ampicillin agar plates. Lipolytic activity was confirmed by incubating the plates at 37 °C for 16 h followed by 48 h incubation at 4 °C. Positive transformants were aseptically picked and cultured overnight at 37 °C in 10 mL LB broth supplemented with a final volume of 50 µg/mL ampicillin. The extracted plasmid was reconfirmed for the presence of the desired lipase gene using double digestion with restriction enzyme (*Bam*H1 and *Xho*1). The optimisation of recombinant protein expression was carried out in three fractions: soluble, intracellular and inclusion body. The T7 expression system allows a high level of expression and leads to efficient transcription of the gene carried on the expression vector [74].

### 4.5. Solubilisation and Refolding of LSK25 Lipase Inclusion Bodies

The recombinant plasmid induced at optimised expression condition was centrifuged (10,000× *g*; 15 min). The pellet obtained was resuspended in 20 mL of Tris-HCl buffer (50 mM; pH 8) and subjected to sonication (output: 2, duty cycle: 20 and 9 min) (Sonifer^®^ SLP150 Branson, Danbury, CT USA). The clear cell lysate obtained after further centrifugation (10,000× *g*; 20 min) contained the soluble protein and the pellet contained the insoluble protein. The pellet obtained after sonication was resuspended in 10 mL Tris-HCl (50 mM; pH 8) containing 6 M urea and incubated for 3 h at 4 °C with constant agitation [18]. After incubation, the suspension was centrifuged (10,000× *g*; 30 min) and renaturation of the supernatant was achieved by a 10× dilution of the denaturant in Tris-HCl buffer (50 mM; pH 8). Solubilized protein was diluted via a dropwise method using a peristaltic pump at 0.5 mL/min and stirred thoroughly at 4 °C for 1 h. The refolded protein was then subjected to lipase assay.

### 4.6. Optimisation of Recombinant Lipase Gene Expression

The LSK25 lipase gene was induced at different isopropyl β-d-1-thiogalactopyranoside (IPTG) concentrations (inducer) ranging from 0 to 2 mM at OD_600_ of 0.5 for 12 h. Optimisation at various growth temperatures (5, 15, 20, 25 30 and 35 °C) was conducted for lipase expression study. Induction of the recombinant clone induced with 0.05 mM IPTG at OD_600_ (0.5) was tested at 4 h intervals over 24 h. This parameter is crucial to determine the incubation time needed for optimal lipolytic activity prior to the addition of IPTG. The cell lysate for each of the optimisation parameters was obtained and subjected to colorimetric lipase assay at a single designated temperature. Molecular weight of LSK25 lipase was determined via sodium dodecyl sulphate-polyacrylamide gel electrophoresis (SDS-PAGE) using 6% stacking gel and 12% separating gel as described by Laemmli (1970) [75]. Electrophoresis was performed at a constant voltage (210 V) (BioRad, Hercules, CA, USA) at room temperature and stained using Coomassie Brilliant Blue R 250 (BioRad, Hercules, CA, USA) for 10 min, and destained with destaining solution. Molecular mass of the protein was estimated using a broad range of protein standard markers (unstained protein marker 18.4–116 kDa, Thermo Scientific, Waltham, MA, USA)

### 4.7. Recombinant LSK25 Lipase Purification

Purification of the His-tagged recombinant lipase was performed using a single step Ni-Sepharose affinity chromatography purification technique. Filtered recombinant crude extract was loaded onto a pre-equlibrated Ni Sepharose^®^ 6Fast Flow column (XK 16/20) (GE, Boston, MA USA). The column was equilibrated with binding buffer [50 mM Tris-HCl, 5 mM imidazole, 500 mM NaCl, (pH 8)] at a flow rate of 1.00 mL/min. The crude protein was loaded onto the column, and ascending step wise gradients of imidazole concentration, ranging from 100 mM to 500 mM were used to elute the bound protein. The bound lipase was eluted using the elution buffer [50 mM Tris-HCl, 500 mM imidazole and 500 mM NaCl (pH 8)], eluted proteins were collected and active fractions containing the protein of interest were confirmed through lipase assay, SDS-PAGE and native-PAGE. Native gel was cast in the absence of sodium dodecyl sulphate (SDS) [76]. Native-PAGE was used to determine the purity and mobility of the purified protein. These fractions were pooled together and stored at 4 °C for further characterization.

### 4.8. Determination of Protein Content

The protein content of the pooled protein was determined by the Bradford method (Bradford, 1976) [77] using bovine serum albumin as the standard. Purified protein concentration was assessed by measuring absorbance at 595 nm.

### 4.9. Activity Staining

Activity staining was performed according to the method outlined by Sommer et al. (1997) [78]. Prior to SDS-PAGE, the gel was immersed in 20% (*v*/*v*) isopropanol for 30 min on a rotary shaker, washed with ultrapure water and overlaid on a tributyrin agar plate. The plate was incubated at 4 °C for 2 h. Activity staining was carried out with a PageRuler Prestained ladder (Thermo Scientific, Waltham, MA, USA).

#### Dynamic Light Scattering (DLS) Analysis of Purified LSK25 Lipase

The dynamic light scattering (DLS) technique is used to determine the size distribution profile of small particles in solution [79]. The measurement of protein particle size in purified LSK lipase at 0.1 mg/mL concentration was carried out using a Zetasizer APS DLS machine (Malvern Panalytical, Malvern, UK), at 830 nm.

### 4.10. Characterization of Purified LSK25 Lipase

#### 4.10.1. Effect of Temperature on Lipase Activity and Stability

Purified LSK25 lipase activity was measured at temperatures ranging from 0 to 55 °C at 5 °C intervals over 30 min. Enzyme thermostability tests were conducted by pre-incubating LSK25 lipase at various temperatures ranging from 0 to 55 °C at intervals of 5 °C for 30 min. The residual activity of all incubated samples was measured at 30 °C (optimum temperature) using olive oil emulsion as the substrate. The stability was determined as the activity relative to the control (unincubated).

#### 4.10.2. Effect of pH on Lipase Activity and Stability

The effect of pH on purified LSK25 lipase activity was investigated by emulsifying the substrate in buffers of different pH ranging from 4.0 to 12.0. The buffers used were: 50 mM sodium acetate (pH 4.0–6.0), 50 mM potassium phosphate (pH 6.0–7.0), 50 mM Tris-HCl (pH 7.0–9.0) and 50 mM glycine-NaOH (pH 9.0–12.0). The effect of pH on lipase stability was investigated by incubating the purified enzyme at various pH values ranging from 4.0 to 12.0 in a 1:1 ratio of buffer with olive oil for 30 min at 25 °C, followed by colorimetric lipase assay. The stability was determined as the activity relative to the control (unincubated). The lipase was assayed at 30 °C.

#### 4.10.3. Effect of Metal Ions on Lipase Activity

Purified LSK25 lipase was treated with 1 mM and 5 mM metal ions individually (Li^+^, Na^+^, K^+^, Rb^2+^, Cs^+^, Mg^2+^, Ca^2+^, Ni^2+^, Mn^2+^, Fe^2+^, Co^2+^, Cu^2+^ and Zn^2+^) for 30 min at 25 °C, followed by colorimetric lipase assay. Further analysis was also carried out using various concentrations (1–15 mM) of Ca^2+^ ions. The stability was determined as the activity relative to the control (without metal ion exposure).

#### 4.10.4. Effect of Natural Oil and Ester Substrates

Natural oils (olive oil, corn oil, sunflower oil, canola oil, mustard oil, coconut oil, rice bran oil, soy oil and palm oil) and ester substrates (*p*-nitrophenyl (Sigma, St. Louis, MO, USA ) (acetate (C_2_), butyrate (C_4_) caprylate (C_8_), decanoate (C_10_), laurate (C_12_), myristate (C_14_) and palmitate (C_16_) were incubated at 25 °C for 30 min. Lipolytic activity of the purified lipase was compared to the activity using *p*-nitrophenyl ester as a substrate. Lipase activity was measured colorimetrically at 30 °C.

#### 4.10.5. Effect of Organic Solvents on Lipase Activity

Purified LSK25 lipase was incubated for 30 min at 25 °C in 11 organic solvents (25% *v*/*v*), selected based on their *log P* values (in parentheses): DMSO (−1.22), methanol (−0.76), acetonitrile (−0.33), ethanol (−0.18), 1-propanol (0.28), benzene (2), toluene (2.5), xylene (3.15), n-hexane (3.6), n-heptane (3.78), and n-hexadecane (8.8). The lipase was assayed colorimetrically at 30 °C and the stability was determined as the activity relative to the control (without organic solvent). *Log P* is defined as the partition coefficient of the measurement of hydrophobicity and hydrophilic phase of solvents [80].

### 4.11. Biophysical Characterisation of Purified LSK25 Lipase

#### Secondary Structure and Thermal Denaturation

The investigation of secondary and tertiary structures of proteins is an important complementary approach to validate protein folding. Circular dichroism (CD) is used for determining the secondary structure and is also routinely used for studying the unfolding and folding of proteins as a function of temperature [72]. Circular dichroism (CD) spectra were recorded using a JASCO J-810 spectropolarimeter (Tokyo, Japan) at 25 °C. LSK25 was dialysed overnight with 10 mM Tris-HCl buffer (pH 8) prior to CD spectral analysis. The secondary structure measurement was conducted in triplicate between wavelengths of 190 to 260 nm using a 1 mm path length and a range of temperatures in order to measure the changes of secondary structure between 5 °C and 70 °C. Results were expressed as molar ellipticity (θ) in milli-degrees. Secondary structure was analysed by using the simple and rapid method of Raussens et al. (2003) [70]. The thermal denaturation (*Tm*) of LSK25 lipase was measured at 222 nm from 5 °C to 70 °C at a 1 °C/min heating rate. *Tm* defined as the midpoint of the sigmoidal melting curve at 0.1 mg/mL purified protein concentration.

## 5. Conclusions

The present study set out to characterize an organic solvent tolerant recombinant cold-adapted lipase, LSK25. The lipase was successfully purified via single-step Ni-Sepharose affinity chromatography, with a purified protein size of 65 kDa. The influence of a range of parameters including temperature, pH, metal ions, natural triglycerides, esters and organic solvents on enzyme activity were studied. Optimum activity was recorded at 30 °C, and unlike most previously reported cold-adapted lipases obtained from various *Pseudomonas* species, LSK25 exhibited optimum pH and highest stability at pH 6. This slightly acidic environmental preference is an unusual feature of this enzyme. This lipase efficiently hydrolysed long chain fatty acids contained in coconut oil and rice brain oil and also *p*-nitrophenyl laurate (C_12_) synthetic ester. The enzyme functioned well in a broad range of generally hydrophobic solvents. Based on the data obtained, this enzyme is an excellent candidate for testing in various biotechnological applications such as food processing, transesterification, agrochemical, pharmaceutical and low temperature industries.
